# Allophilia and Experience: Predictors of Fear in Developing Dementia

**DOI:** 10.1093/geroni/igab046.2307

**Published:** 2021-12-17

**Authors:** Amber Rusch, Michaela Clark, Moyosoreoluwa Jacobs, Carmen Thomassy, Julie Hicks Patrick

**Affiliations:** West Virginia University, Morgantown, West Virginia, United States

## Abstract

Fear of developing dementia is common and has been linked to delays in seeking medical attention (Arlt et al., 2008). We used data from 320 adults (M age = 39.7, SD = 12.3, range 20 – 70) to examine the ways in which objective knowledge and subjective experience with dementia influence positive attitudes toward persons with dementia. We further examined how these constructs related to fear of developing dementia. A path analysis showed the model fit the data well, X2 (DF = 1) = 0.74, p = .39; RMSEA < .001. Objective knowledge and subjective experience were significantly associated with higher allophilia. Allophilia and subjective experiences were associated with personal fear. However, allophilia decreased fear, whereas subjective experiences were associated with increased fear of developing dementia. To clarify these findings, we conducted a moderated regression in which age was examined as a moderator of the relation between allophilia and fear as well as the relation between subjective experience and fear. Significant results were obtained [F (5, 294) = 10.41, p < .001; R2 = .15]. Age moderated the effect of personal experience on fear. Stronger effects emerged for adults in their 20s compared to those in their 40s; similarly, age exerted a stronger effect for those in their 40s than for those in their 50s. Regarding age effects on the relation between allophilia and fear of dementia, for adults in their 20s and 40s, allophilia reduced fear of dementia. For adults in their 50s, allophilia was associated with higher fear.

